# Is Chronic Whiplash-Associated Disorder Associated with Central Nervous System Impairments? A Controlled Observational Study in a Lithuanian Cohort

**DOI:** 10.3390/jcm14176222

**Published:** 2025-09-03

**Authors:** Gintaute Samusyte, Jolita Ciceliene, Evelina Pajediene, Kestutis Stasaitis, Kestutis Petrikonis, Indre Bileviciute-Ljungar

**Affiliations:** 1 Department of Neurology, Medical Academy, Lithuanian University of Health Sciences, 50161 Kaunas, Lithuania; gintaute.samusyte@kaunoklinikos.lt (G.S.); jolita.ciceliene@lsmu.lt (J.C.); evelina.pajediene@lsmu.lt (E.P.); kestutis.petrikonis@lsmu.lt (K.P.); 2 Department of Emergency Medicine, Medical Academy, Lithuanian University of Health Sciences, 50161 Kaunas, Lithuania; kestutis.stasaitis@lsmu.lt; 3 Department of Clinical Sciences (KIDS), Karolinska Institutet, 18288 Stockholm, Sweden; 4 Department of Rehabilitation Medicine, Danderyd University Hospital, 18288 Stockholm, Sweden

**Keywords:** whiplash-associated disorder (WAD), neck pain, disability, traffic collision, sick-leave, cognitive symptoms, memory

## Abstract

**Background/Objectives**: The aim of this study was to investigate the natural course of a whiplash-associated disorder (WAD) in a Lithuanian population with low awareness of the condition. **Methods**: In this controlled observational study, 45 participants, enrolled during the acute period after motor vehicle accident, and 50 matched controls were followed up at 8 months. Clinical evaluation of WAD grades was combined with self-scored questionnaires for pain, WAD symptoms, disability, emotional state, and cognitive impairment. The Quebec Task Force Questionnaire was used to assess persistence or development of new symptoms at follow-up. Demographic and sick leave data were collected. **Results**: The WAD group showed a significant improvement in clinical signs and self-rated scores for pain and disability after 8 months and became largely comparable to the control group. However, only 13 out of 45 WAD participants were symptom-free at follow-up. Persistent neck pain and dizziness/unsteadiness as well as newly developed cognitive complaints were more frequent in the WAD group compared to controls, each reported by around a third of individuals. Logistic regression showed that new cognitive symptoms could be predicted by nausea/vomiting in the acute period and persisting neck pain at follow-up. None of the participants remained on sick leave at follow-up. **Conclusions**: In a country with low awareness of WAD, a larger proportion of individuals remain symptomatic months after acute whiplash injury but maintain their ability to work. The emergence of new cognitive complaints may suggest concomitant central nervous system involvement.

## 1. Introduction

Whiplash-associated disorder (WAD) occurs after a neck/head trauma due to acceleration–deceleration movements of the head followed by extension/flexion of the cervical spine, sustained during a motor vehicle collision [1], but can also be observed in other trauma-related situations such as sports injuries [2] and falls [1]. In earlier studies, the annual incidence rate of WAD was approximately 300–600 cases per 100,000 inhabitants in North America and Western European countries [3]. According to a recent systematic review and meta-analysis, approximately 38% of WAD patients report residual neck pain and headache at 1 year follow-up [4]. The prognostic factors, however, vary between the populations and compensation systems, according to a systematic review [5] and a meta-review [6]. Factors such as age, gender, education, initial degree of pain symptoms and radiculopathies seem not to be clear prognostic factors, neither are post-injury magnetic resonance imaging or radiographic findings [5,6]. Associations between initial pain and anxiety as well as compensation, legal factors, and need for early healthcare have been identified [6]. Furthermore, in an overview of 13 systematic reviews Walton and colleagues found that baseline neck pain intensity and baseline disability have a strong association with the outcome of WAD [7].

Recently, a study using positron emission tomography and computed tomography showed altered uptake of [^11^C]-D-deprenyl correlating with pain intensity and disability during the acute and chronic phases of WAD patients as compared to controls, indicating tissue damage in craniocervical and cervical spine levels [8].

Moreover, several pathways might develop due to initial tissue damage, high pain intensity, and head/neck trauma by itself. A systematic literature review demonstrated evidence for central sensitization mechanisms in chronic whiplash, like persistent pain complaints, hypersensitivity/hyperalgesia/allodynia, etc. [9]. Central sensitization has been shown to be present and correlate with cognitive impairments in WAD patients, comparable to fibromyalgia patients [10]. Vestibular examination by videonystagmography demonstrated vestibular dysfunction due to damage in the cervical region after whiplash [11], which clinically results in dizziness and nausea.

Despite high numbers of road traffic accidents in Lithuania resulting in many injuries (e.g., 3625 during 2010, [12] and 3289 during 2019 [13]), the awareness of chronic WAD is low both among the general public as well as the medical community. As we suggested in our previous article [14], the awareness of WAD could be reflected by the frequency of publications in both social and scientific media. For instance, a search using the keyword “whiplash” on the search engine Google at around the time of the study resulted in only four articles published in Lithuanian webpages during the past 5 years (the latest published in 2010), while a search for “whiplash” in webpages in Swedish language resulted in approximately 60,000 during the same time period in 2014 [14]. Furthermore, an internet search study found no combined interest in “whiplash” and “compensation” among Lithuanian users, as opposed to a strong association observed in countries with a tort system [15]. More than 15 years later, the public awareness in Lithuania remains low, with Google search returning just under 70 entries, a considerable proportion of them on websites offering private healthcare services. Meanwhile, only severe whiplash-associated injuries, resulting in vertebral fractures or subluxations, spinal cord or nerve root damage, are covered by insurance policies.

In our previous study, we investigated the acute clinical WAD symptoms in Lithuanian people seeking help at the emergency department following a motor vehicle accident [14]. This study showed that aside from the most frequent WAD symptoms such as neck and/or shoulder pain, reduced or painful neck movements, headache and dizziness, WAD patients also had significantly higher disability, worse general health status, and higher risk for anxiety and depressive disorders. To investigate a natural course and factors predisposing to development of chronic WAD, the aims of this study were (1) to assess the residual symptoms at least 6 months after a whiplash injury; (2) to investigate if independent collision factors of the accident affected primary outcomes, and (3) to identify if clinical parameters and symptoms during acute and chronic assessments were associated with injury outcomes.

We hypothesized that chronic WAD symptoms would be present at long-term follow-up in approximately 38% of participants, as reported by others [4], resulting in increased disability. An increased rate of sick absence due to remaining symptoms was also expected at follow-up as compared to controls, despite the low awareness of WAD diagnosis in the Lithuanian population.

## 2. Materials and Methods

### 2.1. Participants

A controlled observational cohort study took place in Kaunas, the second largest Lithuanian city of approximately 340,000 inhabitants at the time of the study. Individuals (both drivers and passengers) who searched for medical help after a motor vehicle collision at the Emergency Department of Lithuanian University of Health Sciences Hospital from November 2008 to October 2010 were considered for participation. The main inclusion criteria were the age limit from 18 to 65 years, a motor vehicle accident, and a signed informed consent to participate in the study. Exclusion criteria were major injuries or cervical spine fractures during the accident (confirmed by objective examination and imaging), chronic neurological disorders, previous trauma with residual symptoms, or spine surgery. Individuals who met the selection criteria were invited by phone to visit the Emergency Department of Kaunas University Hospital for clinical examination and completion of questionnaires within 3–14 days after the accident. Every participant underwent a detailed neurological examination by a clinician in order to establish the diagnosis of acute neck distortion. After at least 6 months, the same group of individuals was invited to come back to the Emergency Department for a repeat clinical examination and questionnaires.

As described previously [14], the control group was recruited from acquaintances of the WAD participants or the investigators (mostly from medical personnel and students at the Lithuanian University of Health Sciences (LUHS) in Kaunas, Lithuania). The control subjects who met eligibility criteria were paired with WAD participants one-to-one by choosing the same sex and a similar age, while education and employment status were also considered when possible. Thus, the control group consisted of relatively healthy individuals with no history of a car accident and matched the study group by age, sex, education, and employment status. They underwent an identical physical examination and questionnaires at the time of selection and at least 6 months later.

### 2.2. Objective Examination

To establish the diagnosis of neck distortion and degree of WAD symptoms according to the Quebec Task Force [3], the examination of every individual started with standard muscle strength testing in all groups of upper limb muscles using the Medical Research Council (MRC) scale and deep tendon reflex testing in the upper limbs. Sensory testing was performed in the C2–C7 dermatomal segments using a 10 g monofilament. Point tenderness to palpation was tested at the projections of spinal processes of the cervical and thoracic vertebra as well as paravertebrally. Neck range of motion (ROM) and motion-evoked pain or stiffness were also tested. ROM was assessed by using a metric tape while the subject was comfortably seated on a chair with the hands resting on the thighs. Active neck flexion, extension, rotation, and lateral flexion were assessed by measuring the distance between the two landmarks in neutral position and in maximal motion. The ROM was determined by the difference between the two measures. The landmarks for neck flexion and extension were the sternal notch and the tip of the chin. The landmarks for rotation were the tip of the chin and the lateral tip of the acromion process. The landmarks for lateral flexion were the tip of the mastoid process and the lateral tip of the acromion process [16].

### 2.3. Questionnaires

The following questionnaires and scales were filled out by study participants on both visits:

(1) Visual Analogue Scale (VAS, 0–100 mm)—to assess pain intensity (no pain–worst pain) and general health perception (best health–worst health). Participants were asked to score their overall pain over the last week as well as pain in different regions of the body.
(2) Quebec Task Force Questionnaire (QTFQ)—to assess symptoms before the accident and acute phase (filled out on the first visit) as well as in the chronic phase [17]. The QTFQ was used to determine the development of a) new symptoms after the collision and b) persistence or development of new symptoms during the follow-up period.
(3) Disability Rating Index (DRI)—to evaluate performance in everyday activities [18]. Evaluation of the DRI represents a sum of scores from 12 scales and ranges from 0 to 1200, where higher scores indicate worse functional status.
(4) Cognitive Failure Questionnaire (CFQ)—to measure a person’s likelihood of committing an error in the completion of an everyday task [19]. The CFQ consists of 25 questions, and the sum of all answers ranges from 0 to 100, with the cut-off score of 43 and above reflecting cognitive impairment.
(5) Hospital Anxiety and Depression (HAD) scale—to determine the levels of anxiety and depression that a person is experiencing [20]. The total sum for both HAD anxiety and HAD depression levels ranges from 0 to 21. A score equal to or more than 10 allows suspicion of clinically significant anxiety or depression symptoms.

A validated Lithuanian version of HADS was used [21], while other scales (QTFQ, DRI, and CFQ) were all translated into Lithuanian and backwards by native speakers and readjusted after back translation. After checking the face validity of the questionnaires by three different neurologists, a pilot study of 20 individuals who experienced a vehicle collision was performed. In the pilot group, Cronbach’s alpha was 0.86 for the CFQ and 0.93 for the DRI. Sociodemographic data and collision circumstances were also recorded. Randomization was not applicable in this study due to its observational nature. None of the study assessments were blinded.

### 2.4. WAD Grade Assessment

Information from the interviews, questionnaires, and objective examinations was used to determine WAD grades. In the acute period, WAD grade I was assigned if only complaints were present (including non-neck related new symptoms from the QTFQ); WAD grade II required, in addition, a presence of musculoskeletal signs (pain/tenderness to palpation, painful and/or restricted neck movements); WAD grade III was allocated when neurological impairments were found (abnormalities in at least two domains (i.e., sensation, deep tendon reflexes, muscle power) were required). Participants with none of the above were deemed to have acute WAD grade 0. They did not develop new symptoms during the follow-up and were excluded from further analysis. In the follow-up phase, WAD grades were assigned based on the same criteria. However, chronic WAD grades could not be reliably determined in participants who did not undergo objective examination.

The study protocol was approved by the Kaunas (Lithuania) Regional Bioethics committee (BE-2-57, issued 11 September 2008).

### 2.5. Statistics

Statistical analysis was carried out using the IBM SPSS Statistics 28.0.0.0 software package (IBM Corp., Armonk, NY, USA). The Shapiro–Wilk test was used to check for data normality. For comparisons of continuous variables between and within the groups, parametric tests (Student’s *t*-test, paired samples *t*-test) were used if data were normally distributed (Shapiro–Wilk test, *p* > 0.05), otherwise non-parametric tests (Mann–Whitney U test, Wilcoxon signed ranks test) were employed. For comparisons of nominal data, Pearson’s chi square test was applied (if the expected cell frequencies were less than five, Fisher’s Exact test or Fisher–Freeman–Halton Exact test were used, depending on the number of categories per variable). Stepwise binary logistic regression was used to identify potential predictors of chronic outcomes in the follow-up period. The statistical significance level was set at *p* < 0.05. Data are presented as mean ± standard deviation if normally distributed or as median (interquartile range (IQR) of the 25th and 75th percentile) if non-normally distributed.

## 3. Results

### 3.1. Participants and Factors of Collision

**Figure 1:**
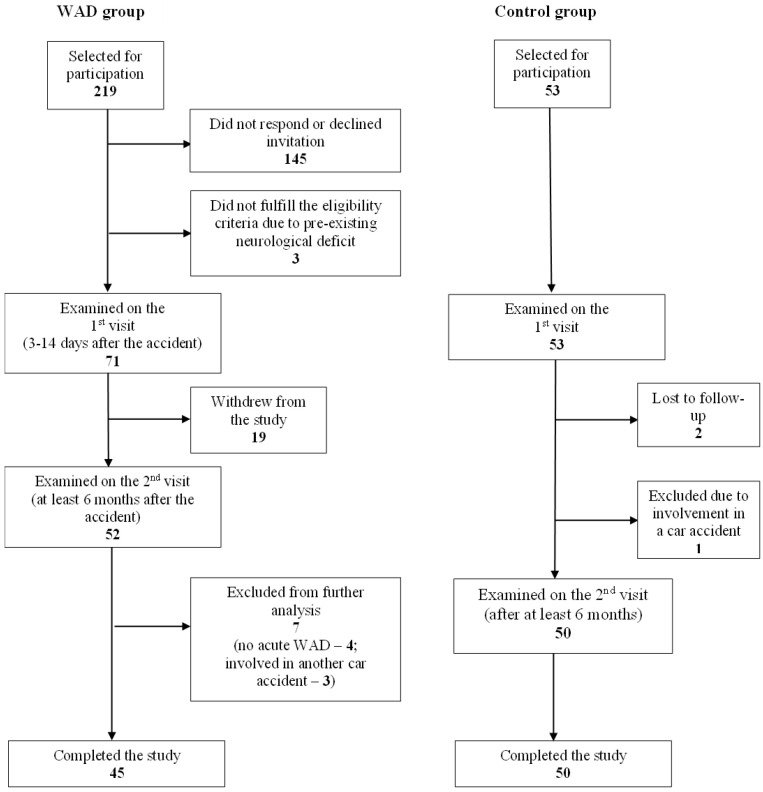
**Participant recruitment flow-chart.**

The participant flow is summarized in Figure 1. Out of 219 individuals who were invited to participate in the study following a motor vehicle collision, 71 were enrolled and examined in the acute period. Fifty-two participants were followed up at least 6 months later, of whom seven were excluded from the final data analysis due to involvement in another car accident (*n* = 3) or absence of WAD (grade 0) in the acute period (*n* = 4). The control group consisted of 53 age- and sex-matched individuals, of whom one was withdrawn from the study due to involvement in a car accident and two were lost to follow-up. Thus, the final WAD and control groups comprised 45 and 50 individuals, respectively. Of note, eight participants in the WAD group and one control subject were unable to attend the study center for the follow-up clinical examination, but returned complete questionnaires.

There were no significant differences in the baseline characteristics (age, BMI, gender, education, and employment) between the study and the control group (Table 1), nor a significant difference in the duration of the follow-up (median 231 (IQR 81) days in WAD group vs. 235 (IQR 86) in control group, Mann–Whitney U test, *p* = 0.87).

Collision circumstances are presented in Supplementary Table S1. The majority of WAD group participants were riding in a car, and more than half of them were drivers. The majority of the accidents were rear-end collisions with no rollover of the vehicle. The median speed during the impact was 50 (IQR 55) km/h. Most participants were sitting in the front of the car, wearing a seat belt, and had a headrest. Approximately 40% of the WAD group subjects sustained trauma to the head, and 20% lost consciousness. Two participants sustained bone fractures (other than spine).

### 3.2. Clinical WAD Grades and Sick Leave in WAD Group

In the acute period, seven (16%) participants had WAD grade I, 36 participants (80%) showed symptoms and signs compatible with WAD grade II, and two participants (4%) had symptoms/signs consistent with WAD grade III. The majority of them (91%) had neck complaints, and only four subjects (all WAD grade I) reported non-neck symptoms in the QTFQ.

At the follow-up, 32 (71%) participants reported at least one complaint in the QTFQ and were considered to have chronic WAD. Of them, eight (25%) had WAD grade I, 15 (47%)—WAD grade II, and three (9%)—WAD grade III, while in six subjects (19%), the grade could not be reliably determined due to missing examination data. Around two-thirds (63%) of participants with chronic symptoms reported neck-related complaints.

**Table 1:** **Baseline characteristics of WAD and control groups.** There were no differences in the baseline demographic characteristics of the study groups. Quantitative variables are presented as mean ± standard deviation if normally distributed or median (25th–75th interquartile range) if non-normally distributed. Qualitative variables are presented as count (group percentage).

	WAD (*n* = 34–45)	Control (*n* = 48–50)	Statistics
**Age, years**	29.5 (19)	28 (16)	Mann–Whitney U test, *p* = 0.961
**Body mass index, kg/m^2^**	23.8 ± 13.3	24 ± 4.3	Student t test, *p* = 0.841
**Sex:**			Pearson‘s chi square test, *p* = 0.858
Men	17 (38%)	18 (36%)	
Women	28 (62%)	32 (64%)	
**Education:**			Pearson‘s chi square test, *p* = 0.428
Secondary	21 (47%)	20 (40%)	
Professional	11 (24%)	10 (20%)	
University	12 (27%)	20 (40%)	
**Employment status:**			Fisher–Freeman–Halton Exact test, *p* = 0.304
Employed	30 (67%)	30 (60%)	
Unemployed	3 (7%)	0	
Not working	2 (4%)	2 (4%)	
Student	8 (18%)	14 (28%)	
Employed student	2 (4%)	4 (8%)	

### 3.3. Specific WAD Symptom Questionnaire (Quebec Task Force Questionnaire, QTFQ)

**Figure 2:**
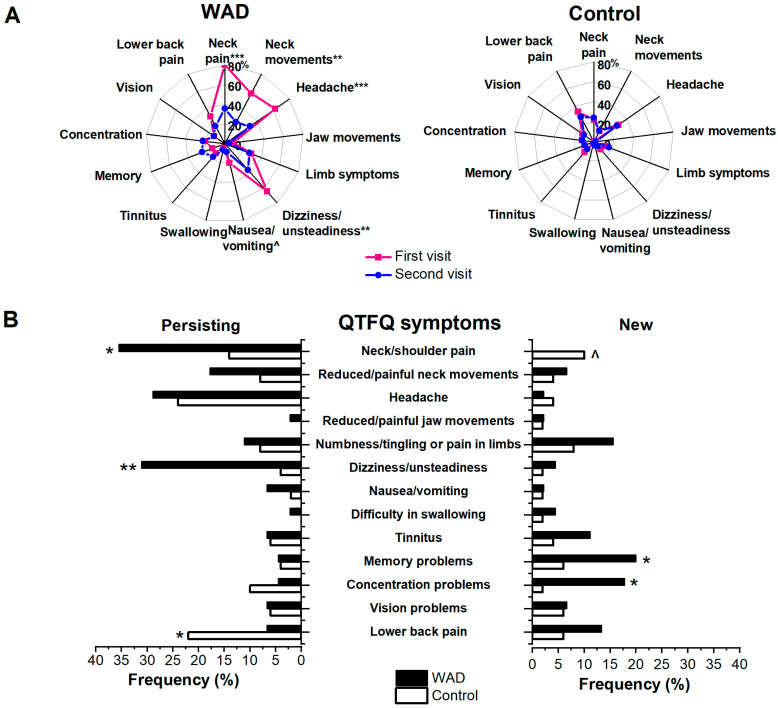
**Symptom frequency in WAD and control groups.** In panel (**A**), the overall symptom frequency (in %) is presented for each group at the initial and the follow-up visits. McNemar’s test was used to assess the change in symptom frequency within each group. As a group, WAD subjects showed a significant recovery in terms of the frequency of neck symptoms, headache, dizziness/unsteadiness, and perhaps nausea/vomiting, while there was no significant change in the frequency of other QTFQ symptoms. Meanwhile, in the control group, the frequency of all QTFQ symptoms remained similar during the follow-up period. In panel (**B**), the WAD and control groups are compared in terms of individual course of symptoms, where persisting symptoms are those reported on both visits, and new symptoms are those reported on the follow-up visit only by the same individual. Chi square test (or Fisher’s Exact test) was used to compare the symptom frequency between the groups. WAD subjects reported more persisting neck pain and dizziness/unsteadiness as well as new cognitive symptoms when compared to the control group, while controls had more persisting lower back pain and tended to develop new neck pain during the follow-up period. *** *p* < 0.0001, ** *p* < 0.001, * *p* < 0.05, ^ *p* < 0.06 (trend). QTFQ—Quebec Task Force Questionnaire.

The QTFQ was used to assess the symptoms experienced by WAD participants in the acute and chronic periods and compare them to the control group (Figure 2). On the initial visit, WAD subjects more frequently reported neck/shoulder pain (χ^2^ = 31.9, *p* < 0.001), reduced/painful neck movements (χ^2^ = 22.2, *p* < 0.001), headache (χ^2^ = 9.9, *p* = 0.002), restricted/painful jaw movements (Fisher’s Exact test, *p* = 0.047), numbness/tingling or pain in the limbs (χ^2^ = 4.2, *p* = 0.04), dizziness/unsteadiness (χ^2^ = 30.6, *p* < 0.001), as well nausea and vomiting (Fisher’s Exact test, *p* = 0.006). Meanwhile, only dizziness/unsteadiness was more common among WAD subjects compared to the controls during the second visit (χ^2^ = 12.9, *p* < 0.001), and a trend for more frequent memory problems was noted (χ^2^ = 3.5, *p* = 0.06).

In addition to comparing overall symptom frequency between the WAD and control groups, the change in symptoms within individual participants was also assessed. Symptoms reported during both visits were considered persistent, while those reported during the second visit only were regarded as newly developed. Even though significantly fewer WAD subjects reported neck symptoms, headache, and dizziness/unsteadiness during the follow-up visit compared to the acute period (Figure 1, panel A), persisting neck/shoulder pain and dizziness/unsteadiness were more common in this group compared to controls (Figure 1, panel B). Although WAD subjects with persisting neck pain reported more overall pain (VAS 34 (IQR 48) vs. 0 (15) mm, Mann–Whitney U test, *p* = 0.010) and worse general health perception (VAS 37 (IQR 43) vs. (12 (IQR 23) mm, Mann–Whitney U test, *p* = 0.022) compared to those without neck pain at follow-up, the DRI, CFQ, and HADS scores did not differ between these subgroups (Mann–Whitney U test, *p* > 0.05). No differences in the scores of the scales were noted between WAD subjects with and without persisting dizziness/unsteadiness (Mann–Whitney U test, *p* > 0.05). There were no significant associations between these persisting symptoms and accident circumstances or acute WAD grade (χ^2^ or Fisher–Freeman–Halton Exact test, *p* > 0.05).

Interestingly, WAD subjects were more likely to develop new memory and concentration problems during the follow-up period compared to controls (Figure 1, panel B). Overall, 29% of WAD subjects reported at least one of these newly developed cognitive complaints compared to 8% of controls (χ^2^ = 7.03, *p* = 0.008). These new cognitive symptoms were not associated with any of the accident circumstances (including trauma to the head or loss of consciousness) or acute WAD grade (χ^2^ or Fisher–Freeman–Halton Exact test, *p* > 0.05). However, significant association was found with tingling/numbness in the limbs and nausea/vomiting reported in the acute period (Fisher’s Exact test, *p* = 0.03 and *p* = 0.001, respectively). The logistic regression model with acute symptoms that were significantly more frequent in the WAD group than in controls (i.e., neck pain and stiffness, headache, restricted/painful jaw movements, limb symptoms, dizziness/unsteadiness, and nausea/vomiting) was significant (χ^2^(7) = 19.9, *p* = 0.006) and explained 51% (Nagelkerke R^2^) of the variance in newly developed cognitive symptoms. However, only nausea/vomiting was a statistically significant predictor of new cognitive complaints in the follow-up period (odds ratio 34.7, 95% confidence interval 2–611).

WAD subjects with new cognitive complaints were more likely to report concomitant persisting symptoms such as neck pain (Fisher’s Exact test, *p* = 0.005), headache (Fisher’s Exact test, *p* = 0.03), numbness/tingling in the limbs (Fisher’s Exact test, *p* = 0.02), and nausea/vomiting (Fisher’s Exact test, *p* = 0.02). Logistic regression with persisting QTFQ symptoms (χ^2^(13) = 24.1, *p* = 0.03, Nagelkerke R^2^ = 60%) showed that only persisting neck pain was a significant predictor of new cognitive complaints at follow-up (odds ratio 11.7, 95% confidence intervals 1.3–109). This was accompanied by higher VAS scores for headache (VAS 0 (22) vs. 0 (0), Mann–Whitney U-test, *p* = 0.02)) and neck pain (VAS 4 (37) vs. 0 (0), Mann–Whitney U-test, *p* = 0.04)), as well as worse general health perception at follow-up (VAS 31 (IQR 33) vs. 12 (IQR 25.75), Mann–Whitney U test, *p* = 0.03)). No differences in other clinical scales were found. Despite the lack of differences in baseline and follow-up CFQ scores (Mann–Whitney U test, *p* > 0.05), a significant worsening of individual CFQ scores was noted in those who developed new cognitive complaints compared to those who did not (increase by 5 (IQR 9.50) points from acute period vs. 0 (IQR 9.75) point change, Mann–Whitney U test, *p* = 0.045)).

Neither persistent neck pain nor dizziness/unsteadiness nor new cognitive complaints were associated with being on sick leave after the accident or its duration.

### 3.4. Parameters of General Health, Pain, Disability, Cognitive Failure, and Depression/Anxiety

In the acute phase, WAD subjects had worse general health perception and VAS pain scores, higher ratings of anxiety, but not depression (as measured by the HADS), and poorer physical and cognitive performance in everyday tasks (as assessed by DRI and CFQ, respectively) compared to the control group (Table 2). The pain scores (last week’s, headache, neck pain) as well as general health perception reported by the WAD group improved significantly during the follow-up period and became comparable to the control group. A marked improvement in physical performance was also noted by WAD subjects, as reflected by the decrease in DRI sum scores. In contrast, CFQ scores did not change significantly during the follow-up period and remained worse compared to controls. Despite significant improvement, HADS Anxiety scores remained greater in the WAD group at follow-up when compared to controls. At follow-up, WAD subjects also scored higher than controls on the HADS Depression subscale.

Nevertheless, it is important to note that despite the differences in median CFQ and HADS Anxiety scores, the rates of clinically significant abnormalities (i.e., scores of ≥43 points and ≥10 points, respectively) were comparable between the groups on both the initial and follow-up visits (Table 2, Chi-squared test, *p* > 0.05).

**Table 2:** **Self-rated scores of general health perception, pain intensity, disability, and cognitive and emotional status on the initial and follow-up visits.** Only a few variables were normally distributed; therefore, for consistency, all data are presented as median (25th–75th interquartile range), and non-parametric tests were used: Mann–Whitney U test for between-group, and Wilcoxon signed ranks test for within-group comparisons (significant *p* values (>0.05) in bold). Data in squared brackets represent number of subjects with clinically significant abnormalities on respective scales. Negative change in scale scores during the follow-up indicates improvement. VAS—Visual Analogue Scale; HADS—Hospital Anxiety and Depression Scale; WAD—whiplash-associated disorders.

Parameter	First Visit	Second Visit	*Within-Group p Value*	Change ([First–Second Visit])
**General health perception (VAS, mm)**				
WAD	32.5 (35)	18 (31)	***0.041***	−4 (35)
Controls	8 (14)	8 (17.25)	*0.155*	1 (10.25)
*Between-group p value*	***<0.001***	*0.051*		***0.012***
**Pain last week (VAS, mm)**				
WAD	49 (38)	8 (33)	***<0.001***	−20 (46)
Controls	4 (16)	5 (19)	*0.489*	0 (12.75)
*Between-group p value*	***<0.001***	*0.456*		***<0.001***
**Headache (VAS, mm)**				
WAD	0 (8)	0 (0)	***0.036***	0 (0)
Controls	0 (0)	0 (0)	*0.270*	0 (0)
*Between-group p value*	***0.018***	*0.569*		*0.074*
**Neck pain (VAS, mm)**				
WAD	23 (61)	0 (2)	***<0.001***	−8 (30.5)
Controls	0 (0)	0 (0)	*0.050*	0 (0)
*Between-group p value*	***<0.001***	*0.202*		***<0.001***
**Lower back pain (VAS, mm)**				
WAD	0 (24)	0 (0)	*0.076*	0 (16.75)
Controls	0 (0)	0 (0.75)	*0.924*	0 (0)
*Between-group p value*	*0.179*	*0.915*		*0.296*
**Disability Rate Index**				
WAD	315 (461.5)	26 (102.5)	***<0.001***	−231 (448)
Controls	20.5 (60.25)	9.5 (49)	*0.857*	0 (24.63)
*Between-group p value*	***<0.001***	*0.109*		***<0.001***
**Cognitive Failure Questionnaire**				
WAD	30 (13) [*n* = 8]	33 (16) [*n* = 7]	*0.308*	3 (13)
Controls	27 (13.25) [*n* = 4]	25.5 (11) [*n* = 7]	*0.565*	−1.5 (11.25)
*Between-group p value*	***0.024***	***0.004***		*0.240*
**HADS Anxiety**				
WAD	7 (4) [*n* = 10]	5 (4) [*n* = 6]	***0.002***	−2 (5)
Controls	4.5 (4) [*n* = 6]	3 (3) [*n* = 6]	***0.002***	−1 (2.5)
*Between-group p value*	***0.002***	***0.002***		*0.400*
**HADS Depression**				
WAD	2 (3) [*n* = 1]	2 (4) [*n* = 0]	*0.596*	0 (2.5)
Controls	1.5 (3) [*n* = 2]	1 (2) [*n* = 2]	***0.044***	0 (1.5)
*Between-group p value*	*0.088*	***0.010***		*0.351*

### 3.5. Neck Range of Motion (ROM)

In the acute period, a significant restriction of neck movement was observed in WAD subjects compared to controls, as assessed by standardized ROM measurements (total sum of 49 (IQR 11) vs. 56 (IQR 11.63) cm, respectively, Mann–Whitney U test, *p* < 0.001)). More precisely, flexion (10 (IQR 5) vs. 13 (IQR 5) cm, Mann–Whitney U test, *p* < 0.001)), left lateroflexion (5 (IQR 2) vs. 7 (IQR 3) cm, Mann–Whitney U test, *p* = 0.001)), and right lateroflexion (5 (IQR 2) vs. 6 (IQR3) cm, Mann–Whitney U test, *p* = 0.002)) were affected in the WAD group, while no significant difference in neck extension and rotation was noted. During the follow-up visit, a significant improvement was noted in flexion (by 1.5 (IQR 4) cm, Wilcoxon Signed Ranks test, *p* = 0.014)), right rotation (by 1(IQR 4) cm, Wilcoxon Signed Ranks test, *p* = 0.021)) and right lateroflexion (by 1(IQR 2) cm, Wilcoxon Signed Ranks test, *p* = 0.026)). However, neck flexion remained somewhat affected in WAD subjects compared to controls (12 (IQR 3) vs. 13 (IQR 3) cm, respectively, Mann–Whitney U test, *p* = 0.005)). However, there were no significant associations between objectively measured neck ROM and subjectively reported neck movement restriction (QTFQ) among WAD subjects, neither in the acute period nor on the follow-up visit (Mann–Whitney U test, *p* > 0.05).

### 3.6. Predicting Chronic WAD

The degree of chronic WAD could not be reliably determined in about a quarter of cases; therefore, WAD group individuals were further divided into those with or without chronic WAD (*n* = 32 and *n* = 13, respectively). Overall, these subgroups were largely similar across most of the subject characteristics as well as performed assessments. There were no associations between chronic WAD and accident circumstances as well as reported pre-/post-accident symptoms in the QTFQ (χ^2^ or Fisher’s Exact test, *p* > 0.05). No significant differences between the chronic and no chronic WAD subgroups were found in the VAS pain scores or other scales or being on sick leave/its duration in the acute phase (Mann–Whitney U test, *p* > 0.05). Meanwhile, in the follow-up period, individuals with chronic WAD reported more neck pain (0 (IQR 25) vs. 0 (IQR 0) as measured by VAS; Mann–Whitney U test, *p* = 0.018)) and showed a trend towards poorer physical performance as assessed by the DRI (39 (IQR 127.5) vs. 0 (IQR 88), Mann–Whitney U test, *p* = 0.06)). Logistic regression analysis, however, did not reveal any significant predictors of chronic WAD (data not presented).

## 4. Discussion

In the late 1990s, a study of a Lithuanian cohort found symptoms following a motor vehicle collision to be brief and self-limiting, with no apparent evolution to chronic WAD [22]. These findings fueled the debate whether public awareness and compensation systems, both lacking in Lithuania at the time of that study, may contribute to the relatively high prevalence of chronic WAD in some Western countries [23]. Motor vehicle insurance became mandatory in Lithuania in 2001 [24], but the public awareness of whiplash injury remained low even a decade later despite high numbers of road traffic accidents [14]. Given that the scope of the first Lithuanian study was rather narrow in terms of WAD symptomatology, we set out to revisit this topic.

### 4.1. Acute WAD

The clinical picture of acute WAD in our cohort is consistent with the literature [25,26], with the severity of injury classified mostly as grade II and neck symptoms (pain and/or restricted movements), headache, and dizziness/unsteadiness as the most frequent complaints. In addition, restricted/painful jaw movements, numbness/tingling, or pain in the limbs as well nausea and vomiting were more frequent in this group compared to controls, while objective assessment of neck range of motion showed restricted forward and lateral flexion. In the acute phase, individuals with WAD reported worse general health perception and pain scores and had higher ratings of anxiety and poorer physical and cognitive performance in everyday tasks compared to the control group.

### 4.2. WAD Symptomatology at Follow-Up

During the follow-up, the WAD group showed a significant improvement both quantitatively and qualitatively in most areas. Despite this, more than two-thirds of WAD subjects remained symptomatic at follow-up (according to the QTFQ), with several characteristics distinguishing them from the control group.

*Neck symptoms and headache.* While neck pain and headache were one of the most common complaints of WAD subjects in the acute phase, their frequency became comparable to that in the control group at follow-up. Quantitatively, WAD subjects showed significant improvement in VAS ratings of overall pain, headache, and neck pain, and their follow-up scores did not differ from those of the control group. These observations are in line with the first Lithuanian whiplash study [22], which found no difference in the frequency or intensity of neck pain and headache in the whiplash group when compared to controls at one year follow-up. However, comparison of groups in this case does not give the full picture of the course of whiplash-related symptoms in individuals. When looking at the individual change in QTFQ symptoms, we found that WAD subjects were more likely to report persisting neck pain (Figure 2B), while the controls tended to develop new neck pain more often during the period of the study. This is not surprising given the high prevalence of neck pain in the general population, with some studies reporting up to 65% of people recovering from an episode at 1 year [27,28]. This may explain the lack of difference in group comparisons when the individual course of symptoms is not taken into consideration in longitudinal studies. The rate of persisting neck pain after whiplash injury in our study was similar to that recently reported in a systematic review and meta-analysis (36% vs. 38%) [4].

Subjective neck stiffness, which was another common WAD complaint in the acute phase paralleled by a significant reduction in the objectively measured neck ROM, improved significantly, and its frequency in the WAD group became comparable to that in the control group. At follow-up, the objectively measured neck forward flexion ROM remained somewhat affected in WAD individuals when compared to controls. However, this was not associated with a subjective perception of neck stiffness.

*Dizziness/unsteadiness.* In our study, dizziness/unsteadiness was one of the most common WAD complaints in the acute phase, reported by more than 60% of individuals. It persisted in about a third of them and remained more frequent at follow-up compared to the control group, which is in line with the literature [29,30]. Persistence of dizziness/unsteadiness was not associated with worse scores on any of the clinical scales.

*Cognitive complaints.* In the acute phase, there was no difference in the frequency of subjective concentration and memory complaints between the WAD and control groups, despite consistently worse CFQ scores in the WAD group throughout the study. Therefore, our finding that WAD subjects were more likely to develop new cognitive complaints during the follow-up period was rather unexpected. Originally, the CFQ, which measures subjective cognitive function, was considered to reflect a personal “trait rather than state”, [19] and its scores were later linked to being more prone to work or car accidents [31]. This could potentially explain why WAD subjects as a group had worse scores with no significant improvement during the follow-up period. Nevertheless, we found a significant worsening in individual CFQ scores in those WAD subjects who reported new cognitive symptoms when compared to those who did not, which substantiates the QTFQ findings.

Memory and concentration problems are more commonly attributed to a traumatic brain injury. Indeed, the rates of self-reported head injury and loss of consciousness during the accident were relatively high in our study (40% and 20%, respectively), but these collision-related factors were not linked to the development of new cognitive symptoms in our cohort, nor was the presence of headache in the acute phase. Of all acute symptoms, only tingling/numbness in the limbs and nausea/vomiting showed an association, while only nausea/vomiting was found to be a significant predictor of new cognitive symptoms at follow-up. WAD subjects with new cognitive complaints were also likely to report persistence of neck pain, headache, numbness/tingling in the limbs, and nausea/vomiting, with persisting neck pain being a significant predictor of new cognitive symptoms in a logistic regression model. This was accompanied by worse VAS scores for headache and neck pain as well as general health perception. However, overall pain intensity was low-moderate during the first visit and low during the second visit, indicating low probability of central sensitization or chronic pain disorder by itself and possibly absence of clinical relevance in statistical prediction models. Moreover, the latest evidence indicates that central sensitization might be related rather to psychosocial factors than central nervous mechanisms when using the Central Sensitization Inventory [32] and quantitative sensory testing (QST) [33]. Meanwhile, the QST findings on hyperalgesia during the acute phase could predict recovery after 3 months or later, according to a systematic review [34].

Vestibular dysfunction has also been linked to the development of cognitive impairment in some conditions [35], though in the context of whiplash injury, this relationship is yet to be determined. While persisting dizziness/unsteadiness was common in our cohort, we found no associations with new cognitive complaints.

*Anxiety.* Psychological disturbances, such as anxiety and depression, have been found to be more common among WAD subjects than in the general population [36]. In our study, WAD subjects had higher HADS Anxiety scores both initially and at follow-up, although the frequency of clinically significant abnormalities was comparable between the groups at both visits. While in the acute period, this could be attributed to an acute stress reaction, persistence of physical symptoms such as neck pain and headache has been proposed as a contributing factor in the chronic period [36]. We found no associations between persisting or newly developed WAD symptoms and anxiety or depression scores at follow-up.

*Whiplash or concussion?* In a general clinical setting not specialized in managing WAD patients, only neck symptoms would likely be considered a direct consequence of whiplash injury to cervical tissues, while nausea, dizziness, and particularly cognitive complaints months after a car accident would prompt clinicians to consider a mild traumatic brain injury. Studies on chronic WAD suggest cervical muscle fat infiltration as a marker of severe WAD [37,38]. Recent studies indicate a link between this biomarker and lower brain network in WAD patients after 3 months, suggesting a presence of brain functional abnormalities [39]. Thus, the combination of the above-described follow-up findings raises the question of whether the development of cognitive symptoms could be partly attributed to a concomitant traumatic brain injury or if persisting neck symptoms may have led to worsening cognitive functions without a direct brain injury.

The link between chronic pain conditions and declines in neurocognitive functions, including attention and memory, has been well-described [40]. Pain intensity has also been linked to worse cognitive functioning in whiplash patients [41]. Therefore, on one hand, one might speculate that chronicity of neck pain may have led to the development of new cognitive symptoms in our WAD cohort, especially given that they were not associated with accident circumstances suggestive of potential brain injury. Although pain persisted in 16 out of 45 participants, neck pain intensity was low (VAS 8/100) at follow-up, indicating absence of pain spreading in the cervical spine structures. Secondary factors, such as sleep disturbance [42] or use of pain medication [43], may play a role, but they have not been addressed in this study.

On the other hand, the overlap between whiplash and concussion has been long recognized [28]. Linear accelerations resulting in concussion are well above the thresholds for mild strain injuries of the neck [44], which would explain why neck symptoms are common after concussion [45]. Neck pain has also been shown to predict poorer post-concussion outcomes [45,46,47]. Conversely, biomechanical studies suggest that even small kinetic shocks that cause whiplash may exert strain on the brain sufficient to induce mild injury, especially if the head is rotated [48]. While routine brain imaging is negative in whiplash injury, subtle impairment can be detected by advanced techniques. For example, diffusion tensor imaging demonstrated diffuse axonal lesions in whiplash patients, neuroanatomically corresponding to their symptoms [49], while resting-state functional magnetic resonance imaging revealed a reduction in brain network modularity, which is also observed in persisting post concussive syndrome [39]. These observations, albeit from a very limited number of studies, could somewhat explain the overlap between the two conditions in terms of symptomatology and outcomes [43,50].

*Sick leave.* Up to around 20% of individuals report reduced work capacity due to persistent symptoms following whiplash injury [51], and cognitive complaints were found to be an independent predictor for these long-term issues [52]. In our cohort, none of the WAD subjects remained on sick leave for an extended period that would prompt formal assessment of long-term incapacity to work, nor did they have to change their employment status due to health issues. This observation further supports the notion that medicolegal and psychosocial factors likely play a role in the long-term disability after whiplash injury [6].

### 4.3. Strengths and Limitations

The first Lithuanian whiplash report that contributed to the controversies in the field [22] was questionnaire-based with a very narrow scope in terms of WAD presentation and conducted by mail. Our study investigated the broad spectrum of WAD symptomatology and its potential impact on individual physical and cognitive performance as well as emotional state by means of standardized clinical scales and objective examination, all carried out by trained physicians. The study also includes matched controls followed with the same measurements which is important in comparative studies since a global prevalence for neck pain during that period was approximately 21% [53]. Despite this, several important limitations should be mentioned. Firstly, the study enrolled only those individuals who visited the emergency department following a motor-vehicle collision in a single regional (university) hospital. The initial response rate (around 30%) was rather low with further dropouts during the follow-up period or incomplete assessments on the second visit, resulting in a relatively small sample size. This may have biased the study towards the inclusion of subjects with more severe injuries and initial symptoms. Secondly, the clinical scales used in this study, although standardized, were all based on self-reporting. While a recent meta-analysis showed that CFQ subjective scores can predict objective performance on some executive function tasks [31], formal neuropsychological testing would have been preferable to corroborate subjects’ cognitive complaints. Thirdly, although our findings are generally in line with the literature, the lack of associations with previously established risk factors for chronic WAD may be due to a relatively small sample size, especially when considering WAD subgroups in the chronic period, which limits generalizability. Lastly, in light of considerable socioeconomic changes that Lithuania has undergone since the completion of the study, a delay in reporting may seemingly diminish the relevance of our findings. However, the public awareness of WAD remains low and compensations systems are still unfavourable. Therefore, we believe that our results remain important not only because they contrast the first widely cited Lithuanian study on whiplash [22], but also due to a somewhat unexpected key finding, i.e. rather frequent new cognitive complaints during the follow-up. 

## 5. Conclusions

In this small Lithuanian cohort, the clinical picture and severity of acute WAD was comparable to that described in the literature. Despite significant quantitative and qualitative improvements after 8 months, more than two-thirds of WAD subjects remained symptomatic, with persisting neck pain and dizziness/unsteadiness as well as newly developed cognitive complaints, which were more frequent than in the control group. This, however, did not affect their employment status. The emergence of subjective cognitive disturbances, which could be predicted by nausea/vomiting in the acute period and persisting neck pain at follow-up, points to the possibility of a concomitant brain injury.

## Data Availability

The datasets presented in this article are not readily available because of ethical and legal restrictions that were in place at the time of the study.

## Supplementary Materials

The following supporting information can be downloaded at: https://www.mdpi.com/article/10.3390/jcm14176222/s1, Table S1: Collision circumstances. File S1: TROBE Statement—checklist of items that should be included in reports of observational studies.
