# Supplementary material for: Is Chronic Whiplash-Associated Disorder Associated with Central Nervous System Impairments? A Controlled Observational Study in a Lithuanian Cohort
Source: J Clin Med. 2025 Sep 3;14(17):6222. doi: 10.3390/jcm14176222 (PMC12429458; doi:10.3390/jcm14176222)
Supplement: Supplementary file 1 [file jcm-14-06222-s001.zip › jcm-3750445-supplementary File S1.pdf]

STROBE Statement—checklist of items that should be included in reports of observational studies

	Item No.	Recommendation	Page # where this item is located:
**Title and abstract**	1	(*a*) Indicate the study's design with a commonly used term in the title or the abstract	p. 1, line 2 and 18.
		(*b*) Provide in the abstract an informative and balanced summary of what was done and what was found	p. 1, lines 16-31.
**Introduction**			
Background/rationale	2	Explain the scientific background and rationale for the investigation being reported	p. 2, lines 69-88, p. 5, lines 104-107
Objectives	3	State specific objectives, including any prespecified hypotheses	p. 2 lines 88-96
**Methods**			
Study design	4	Present key elements of study design early in the paper	p. 2, line 100.
Setting	5	Describe the setting, locations, and relevant dates, including periods of recruitment, exposure, follow-up, and data collection	p. 2-3, lines 101- 104, p. 3, lines 110-115.
Participants	6	(*a*) *Cohort study*—Give the eligibility criteria, and the sources and methods of selection of participants. Describe methods of follow-up<br>*Case-control study*—Give the eligibility criteria, and the sources and methods of case ascertainment and control selection. Give the rationale for the choice of cases and controls<br>*Cross-sectional study*—Give the eligibility criteria, and the sources and methods of selection of participants	p. 2-3, lines 104-115.
		(*b*) *Cohort study*—For matched studies, give matching criteria and number of exposed and unexposed	p. 3, lines 116-125.

		Case-control study—For matched studies, give matching criteria and the number of controls per case	
Variables	7	Clearly define all outcomes, exposures, predictors, potential confounders, and effect modifiers. Give diagnostic criteria, if applicable	P. 3, lines 127-189.
Data sources/ measurement	8*	For each variable of interest, give sources of data and details of methods of assessment (measurement). Describe comparability of assessment methods if there is more than one group	p. 3, lines 127-189.
Bias	9	Describe any efforts to address potential sources of bias	p. 4, lines 1218-220.
Study size	10	Explain how the study size was arrived at	Study size was not calculated, since there was no data on whiplash injury in Lithuania.

Continued on next page

Quantitative variables	11	Explain how quantitative variables were handled in the analyses. If applicable, describe which groupings were chosen and why	Cohort was divided into groups whiplash participants and matched controls. More information on controls, please, see p. 3, lines 116-125.
Statistical methods	12	(*a*) Describe all statistical methods, including those used to control for confounding	The statistics is done for comparision between the two groups and within follow-up time, tests were chosen according distribution of sample and the nature of each variable. Logistic regression was used to identify the predictors.
		(*b*) Describe any methods used to examine subgroups and interactions	
		(*c*) Explain how missing data were addressed	Missing data for clinical variables is reported for those participants who did not arrive to follow-up but send in questionnaires. Please, see p. 4, lines 218-220.
		(*d*) *Cohort study*—If applicable, explain how loss to follow-up was addressed<br>*Case-control study*—If applicable, explain how matching of cases and controls was addressed<br>*Cross-sectional study*—If applicable, describe analytical methods taking account of sampling strategy	
		(*e*) Describe any sensitivity analyses	
**Results**			

Participants	13*	(a) Report numbers of individuals at each stage of study—eg numbers potentially eligible, examined for eligibility, confirmed eligible, included in the study, completing follow-up, and analysed	
		(b) Give reasons for non-participation at each stage	
		(c) Consider use of a flow diagram	Figure 1.
Descriptive data	14*	(a) Give characteristics of study participants (eg demographic, clinical, social) and information on exposures and potential confounders	Table 1.
		(b) Indicate number of participants with missing data for each variable of interest	Number of participants for each variable is indicated in Table 1.
		(c) *Cohort study*—Summarise follow-up time (eg, average and total amount)	P. 4, lines 223-224.
Outcome data	15*	*Cohort study*—Report numbers of outcome events or summary measures over time	Table 1-2 and Figure 2.
		Case-control study—Report numbers in each exposure category, or summary measures of exposure	
		Cross-sectional study—Report numbers of outcome events or summary measures	Presented in Tables and Figures.
Main results	16	(*a*) Give unadjusted estimates and, if applicable, confounder-adjusted estimates and their precision (eg, 95% confidence interval). Make clear which confounders were adjusted for and why they were included	Regression analysis with estimates is presented in Results, Section 3.6.
		(*b*) Report category boundaries when continuous variables were categorized	
		(*c*) If relevant, consider translating estimates of relative risk into absolute risk for a meaningful time period	

Continued on next page

Other analyses	17	Report other analyses done—eg analyses of subgroups and interactions, and sensitivity analyses	
**Discussion**			
Key results	18	Summarise key results with reference to study objectives	p. 11-13., lines 441-445, p. 12, lines 457-459, 466-469, 473-475, 482- 484,491-492, 504-511, 544-546 and 565-568.
Limitations	19	Discuss limitations of the study, taking into account sources of potential bias or imprecision. Discuss both direction and magnitude of any potential bias	p. 13-14, lines 573-591.
Interpretation	20	Give a cautious overall interpretation of results considering objectives, limitations, multiplicity of analyses, results from similar studies, and other relevant evidence	p.14, lines 581-585 and 588-591.
Generalisability	21	Discuss the generalisability (external validity) of the study results	p. 14, lines 592.
**Other information**			
Funding	22	Give the source of funding and the role of the funders for the present study and, if applicable, for the original study on which the present article is based	p. 14, line 624-625.
