# Supplementary material for: Is Chronic Whiplash-Associated Disorder Associated with Central Nervous System Impairments? A Controlled Observational Study in a Lithuanian Cohort
Source: J Clin Med. 2025 Sep 3;14(17):6222. doi: 10.3390/jcm14176222 (PMC12429458; doi:10.3390/jcm14176222)
Supplement: Supplementary file 1 [file jcm-14-06222-s001.zip › jcm-3750445-supplementary.pdf]

**Supplemental Table S1. Collision circumstances.**

| Reported circumstances | n (%) |
|---|---|
| **Vehicle:** | |
| Car | 43 (96%) |
| Bus | 2 (4%) |
| **Collision:** | |
| Front | 9 (20%) |
| Back | 19 (42%) |
| Driver´s side | 8 (18%) |
| Passenger´s side | 9 (20%) |
| **Seat in the vehicle:** | |
| Driver | 27 (60%) |
| Passenger in front seat | 10 (22%) |
| Passenger back left | 2 (4%) |
| Passenger back right | 4 (9%) |
| Passenger back middle | 2 (4%) |
| **Seat belt:** | |
| Yes | 32 (71%) |
| No | 10 (22%) |
| Could not answer | 3 (7%) |
| **Headrest:** | |
| Yes | 34 (76%) |
| No | 11 (24%) |
| **Remained in the seat:** | |
| Yes | 8 (18%) |
| No | 34 (76%) |
| Could not answer | 3 (7%) |
| **Rollover:** | |
| Yes | 2 (4%) |
| No | 43 (96%) |
| **Lost consciousness:** | |
| Yes | 9 (20%) |
| No | 36 (80%) |
| **Trauma to the head:** | |
| Yes | 18 (40%) |
| No | 24 (53%) |
| Could not answer | 3 (7%) |
| **Fractures (other than spine):** | |
| Yes | 2 (4%) |
| No | 41 (91%) |
| Could not answer | 1 (2%) |
